# Book Review

**DOI:** 10.1120/jacmp.v7i2.2239

**Published:** 2006-05-25

**Authors:** Michael G. Herman

**Affiliations:** ^1^ Vice Chairman and Head of Physics Mayo Clinic Rochester Minnesota 55905 U.S.A.

## Abstract

*The Modern Technology of Radiation Oncology*, Vol. 2, edited by Jacob Van Dyk, Medical Physics Publishing, 2005, ISBN‐10:1‐930524‐25‐0 (hard cover), list price $120 hard cover, $100 soft cover

*The Modern Technology of Radiation Oncology*, volume 1, is a compendium of the technology and techniques and their implementation in modern radiation therapy practice, and was published in 1999. Since that time, a number of developments and enhancements to the practice of radiation oncology have been undertaken. *The Modern Technology of Radiation Oncology*, volume 2, updates and further details some technologies that were introduced in volume 1; it also introduces in detail technologies that were too new or nonexistent and not reviewed in volume 1. For example, Chapter 2, Imaging and Radiation Therapy Planning, is a followup and enhancement to volume 1's Chapter 7 on a similar topic. Likewise, Chapter 10, Prostate Brachytherapy, is a detailed expansion of this subject, only introduced in volume 1, Chapter 18. Overall, this describes the significant incremental advances in radiation therapy technology since volume 1 was published a few years ago. This book is intended for, and likely useful for, medical physicists at all levels in their careers, medical physics residents, and, to some extent, medical residents for some applications. Certain aspects of the book would also be useful for dosimetrists and radiation therapists in training and during their practice.

Chapter 1 presents the motivating summary for this volume, referring to new technology that allows higher, more conformal radiation doses to be delivered. Each new technology requires additional understanding, quality assurance, precision, accuracy, etc. Of great significance in this very complex technological environment are the issues of quality assurance relative to reducing errors. It is also important to recognize that new technologies present new challenges, and these challenges may impact the clinical result. What we do now for new techniques should not be what we have always done because the risks and failure modes may be different. This at times requires an assessment to improve the process with new technology, even if it means significant change.

Chapter 2 updates the reader on imaging for treatment planning. It includes accounting for motion and four‐dimensional scanning as well as updates on MR, PET, PET CT, and SPECT. A detailed review of the Task Group 66 Report on CT simulation is also included, although it might be too detailed because TG 66 is a published report.

Chapter 3 is an excellent introduction to Monte Carlo treatment‐planning solutions, but it may be too detailed on some of the technical aspects, which makes locating critical and important information difficult for the average reader. Good clinical examples are shown.

Chapter 4 is a detailed and thorough description of inverse planning with specific and clinically meaningful examples. It pays particular attention to the nuances that physicists and dosimetrists need to understand to effectively use inverse planning algorithms. This includes understanding how the system behaves with variations in key parameters, objectives, constraints, prioritizations, and prescriptions. This chapter also goes into a lot of detail rather than leaving anything out.

Chapter 5 is an overview of and introduction to the development of current radiobiological models and their limitations. It points out the real lack of good experimental data to build reliable and predictable radiobiological models. The history of the linear quadratic model and the derivation of tumor control probability and normal tissue complication probabilities are described. Existing data are reviewed in the context of the models and their limitations. The authors stress the true complexity in developing a model with minimal data that are meaningful when extrapolated to radiation therapy treatments.

Chapter 6 presents an update on intensity‐modulated radiotherapy (IMRT), but it is somewhat redundant with Chapter 4 in this volume and Chapter 12 of volume 1. This, to some extent, is confusing to the reader because the nomenclature is slightly different in this chapter than in the previous discussions of IMRT. For the reader to jump back and forth between Chapters 4 and 6 of this volume and Chapter 12 of the previous volume is too complex. In addition, the use of vendor‐specific examples may not be generally applicable to all users.

Chapter 7 updates the radiographic verification process and reviews advanced solutions for this process, namely, image‐guided radiation therapy. It includes the essential procedures for the use, commissioning, and quality assurance of kilovoltage, megavoltage, cone‐beam CT, and CT‐based methods of localization. It drives home the point that the treatment plan is intended to deliver a specific dose to the target, and that the verification protocols allow the team to be certain, through an organized technical process, that the dose was indeed delivered as intended.

Chapter 8 introduces and brings the reader quickly up‐to‐date on the status of breathing and breathing control in radiation therapy treatments. This is a very good review of the current state of respiratory issues and their potential solutions.

Chapter 9 describes the essentials and basics related to the use of ion chambers for absolute calibration of megavoltage beams. Some of the basics overlap with those already considered in volume 1 on dose measurement tools, specifically ion chambers. This is, however, a very good summary of currently applicable calibration protocols. It also underscores, and clearly covers, the critical importance of quality assurance of calibration procedures.

Chapter 10 updates the brachytherapy practice, specifically for prostate implants, and includes a current and substantial review of low‐dose rate and high‐dose rate techniques for prostate brachytherapy.

In summary, volume 2 of *The Modern Technology of Radiation Oncology* contains some very useful information for medical physicists at any stage in their career, for radiation oncology residents, and for dosimetrists and radiation therapists. Although some of the information is too detailed and some is redundant with volume 1 and with other chapters of this volume, most would find this a reference worth having in their library.

